# FinKENet: A Novel Financial Knowledge Enhanced Network for Financial Question Matching

**DOI:** 10.3390/e26010026

**Published:** 2023-12-26

**Authors:** Yu Guo, Ting Liang, Zhongpu Chen, Binchen Yang, Jun Wang, Yu Zhao

**Affiliations:** 1 Financial Intelligence and Financial Engineering Key Laboratory of Sichuan Province, Fintech Innovation Center, Southwestern University of Finance and Economics, Chengdu 611130, China; guoyugy@smail.swufe.edu.cn (Y.G.); zpchen@swufe.edu.cn (Z.C.); yangbc@smail.swufe.edu.cn (B.Y.); 2 School of Accounting, Southwestern University of Finance and Economics, Chengdu 611130, China; liangt@swufe.edu.cn; 3 School of Management Science and Engineering, Southwestern University of Finance and Economics, Chengdu 611130, China

**Keywords:** question matching, siamese neural network, attention mechanism, cross-entropy

## Abstract

Question matching is the fundamental task in retrieval-based dialogue systems which assesses the similarity between Query and Question. Unfortunately, existing methods focus on improving the accuracy of text similarity in the general domain, without adaptation to the financial domain. Financial question matching has two critical issues: (1) How to accurately model the contextual representation of a financial sentence? (2) How to accurately represent financial key phrases in an utterance? To address these issues, this paper proposes a novel **Fin**ancial **K**nowledge **E**nhanced **Net**work **(FinKENet)** that significantly injects financial knowledge into contextual text. Specifically, we propose a multi-level encoder to extract both sentence-level features and financial phrase-level features, which can more accurately represent sentences and financial phrases. Furthermore, we propose a financial co-attention adapter to combine sentence features and financial keyword features. Finally, we design a multi-level similarity decoder to calculate the similarity between queries and questions. In addition, a cross-entropy-based loss function is presented for model optimization. Experimental results demonstrate the effectiveness of the proposed method on the Ant Financial question matching dataset. In particular, the Recall score improves from 73.21% to 74.90% (1.69% absolute).

## 1. Introduction

**Figure 1:**
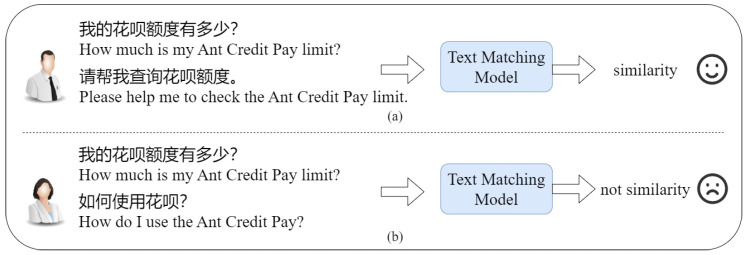
**Cases of financial question-matching task**. (**a**) is a similar utterance-pairs, (**b**) is not a similar utterance-pairs.

Recently, with the emergence of ChatGPT [1], artificial intelligence has once again become a hot topic for the public. ChatGPT has a powerful general language capability that can assist in various aspects of human daily life and work. In the Chinese Natural Language Processing (NLP) domain, there are also products like ChatGLM [2,3], ERNIE Bot [4], and MOSS (MOSS: https://github.com/OpenLMLab/MOSS) playing important roles. These dialogue systems fine-tuned from large language models demonstrate significant capabilities in the general domain. When they are applied to vertical domains such as healthcare and finance, further fine-tuning and refinement are needed. However, fine-tuning a large language model is costly work. Therefore, for vertical domains, using retrieval-based dialogue systems is a viable choice. Question-matching technology is a critical technique for retrieval-based dialogue systems [5,6]. It calculates the similarity between a user query and a set of predefined questions, returning the top-k questions with the highest similarity scores. These questions are then presented for the system to further select from for response. In the NLP domain, question matching is regarded as a text-matching task, specifically a binary classification task. As shown in Figure 1, given two input texts, the target is to output a label indicating whether they are similar or dissimilar. It can also be viewed as a text similarity calculation task, where the objective is to input two texts and compute the similarity between them. If the computed similarity exceeds a predefined threshold, the texts are considered similar; otherwise, they are considered dissimilar.

Previous methods often treat the question-matching task as a text similarity-matching task. Huang et al. [6] introduced a deep structured latent semantic method (DSSM) that models queries and documents in a shared Euclidean space to calculate the similarity between documents and user queries. Shen et al. [5] improved the DSSM by using convolutional neural networks (CNN) to model the low-dimensional semantic vector space of search user queries and documents. Pang et al. [7] transformed the text classification problem into an image classification problem via stacking CNN modules to extract features. These methods achieve high accuracy of text similarity tasks in general domains, but they exhibit poor generalization performance in vertical domains.

In this paper, we focus on financial question matching. Some studies investigate how to integrate text-matching techniques in financial domains. For instance, Tan et al. [8] employed a hybrid model consisting of CNN and RNN to model long sequential text for insurance domain question-answering tasks. Li et al. [9] introduced a text-matching technique specifically for risk guarantees, addressing the gaps in the risk advisory community. Although these methods show some applicability in the financial domain, due to the specialty of such a field, further customization is necessary. Firstly, context is highly important in the financial NLP domain. For instance, in the investment market, stock prices can fluctuate based on contextual influences. Secondly, the financial domain abounds with specialized terminology, which is unfamiliar to the general NLP domain, adaptation training is required to acquaint the model. Table 1 displays various common financial phrases in everyday Chinese language, all sourced from a Chinese financial problem-matching dataset (Section 4.1). The modeling of financial text context and the ability to recognize financial phrases both significantly impact the inferential capabilities of model. Therefore, designing a model that can effectively adapt to financial question matching become one of the most crucial tasks at present. In particular, the design of the financial question matching model faces **two key challenges**: **(1) How to model a contextual utterance more accurately in the financial domain? (2) How to represent financial phrases more accurately?**

To address these challenges, we propose a novel Financial Knowledge Enhanced Network (FinKENet) that incorporates financial knowledge into text representations. We consider the uniqueness of text in the financial domain, the proposed model is designed to lean more towards representing financial text. Firstly, we design a multi-level encoder layer, including sentence-level and phrase-level representation. Specifically, the sentence-level representation aims to encode financial text representations that make them biased toward the financial context. To this end, the proposed model utilizes FinBERT [10] to encode text vectors. Then, the phrase-level representation is able to enhance the adaptability of utterances to financial contexts by directly encoding financial phrases within sentences. Additionally, to facilitate the fusion of sentence vectors and financial keyword phrase vectors, the proposed model utilizes a financial co-attention adapter which can fuse both from sentence to phrase and from phrase to sentence. Finally, we design a multi-level similarity decoder layer to comprehensively predict the similarity between query and question from three perspectives (cosine similarity, manhattan distance, euclidean distance), enhancing the generalization capabilities. In contrast to the additional knowledge injection [11] in language models like GPT and BERT, our proposed model focuses on the fusion process of knowledge.

We present a Cross-entropy-based objective function for training all model parameters. Experimental results on the Ant Financial Question Matching Corpus (AFQMC) show that the proposed FinKENet surpasses all previous baseline models, and becomes the new state-of-the-art (SOTA) model. Since the FinKENet effectively models the financial context, it fills the gaps in this domain. The main contributions of this work are as follows:

- We introduce a novel financial knowledge-enhanced network that explicitly incorporates financial knowledge into text representations, which have a multi-level encoder layer consisting of sentence-level representation and phrase-level representation.
- Specifically, we propose a financial co-attention adapter, which extracts attention vectors from both sentences to phrase and from phrase to sentence, thereby enhancing the text representation capabilities of the method.
- We introduce a multi-level similarity decoder layer that enhances the discriminative power of the model from three perspectives.
- Experimental results demonstrate that the proposed model performs significantly better than the previous state-of-the-art (SOTA) model.

The remaining sections of this paper are organized as follows: Section 2 introduces the related work on dialogue systems and question matching. Section 3 introduces the implementation principles and technical details of the proposed model. Section 4 introduces the experimental design and analyzes the experimental results. Section 5 introduces the analysis of ablation experiments, analysis of the multi-level similarity decoder, and analysis of the case study. Section 6 summarizes the paper and discusses the future research directions of the work.

## 2. Related Work

### 2.1. Dialogue Systems

Dialogue systems have gained significant attention as a research focal point with the advancement of natural language processing technology and artificial intelligence. In the technical implementations of dialogue systems, research mainly focuses on retrieval-based and generative-based methods. Retrieval-based methods use predefined rules and patterns to match user input questions and perform semantic parsing to determine the question’s intent and answer. This approach can address some simple questions but struggles with complex questions and diverse expressions. Al-Ajmi et al. (2021) introduced a hybrid approach that combines rule-based and data-driven methods to construct a text-based system for booking flights through dialogue. By incorporating the Wit.ai natural language interface and Wizard of Oz technology for dialogue flow configuration, the system demonstrates effective comprehension of user inputs and autonomous adaptation. Another study [12] focused on the impact of user personality on the task performance of rule-based dialogue systems. Researchers used a dialogue system based on the MultiWOZ task. They found outgoing and friendly people tend to perform poorly on the task, while neurotic people are more prone to accomplish the task successfully. The study revealed that average utterance length and fillers per sentence, two key dialogue behavior features, have a strong association with how well users complete the task and their personality traits. In addition, Niimi et al. [13] proposed a rule-based method for extracting dialogue acts and topics. This approach was applied to a task-independent spoken dialogue system SDSKIT-3, which controls dialogues in different task domains by using task-related topic frameworks and manually designed utterance analysis rules. This suggests that rule-based methods have some effectiveness in specific task domains.

Currently, research in the field of dialogue systems is primarily focused on improving design details to enhance the practical application value of dialogue systems in various fields. To better address the dialogue challenges in closed domains, researchers have developed some domain-specific dialogue system frameworks. Nakano et al. [14] introduced the HRIChat framework, which supports domain-related language understanding and integrates multiple dialogue management approaches, making the dialogue system outstanding in handling domain-specific discourses. These achievements show that focusing on the research of dialogue systems in closed domains has great potential. To solve the challenges of dialogue system design in dynamic systems, scholars have explored new design ideas and methods. Alty [15] suggested that human-centered design and knowledge-based dialogue architecture can effectively improve the flexibility and structured degree of dialogue systems. These studies emphasize the importance of considering user needs and interactive experience when designing dialogue systems. Ultes et al. [16] presented PyDial, a freely available end-to-end statistical spoken conversation system toolkit that supplies implementations of statistical approaches relevant to all components of a dialogue system. In addition, the toolkit has been expanded to offer multi-domain conversation capabilities, with simple configuration, easy expandability, and domain-independent implementations of each dialogue system module. Advanced techniques such as multi-task learning and graph attention networks have also been introduced into knowledge dialogue system research. Zhao et al. [17] put forward a multi-task learning framework built on graph attention networks intended for multi-domain goal-driven conversational systems. The introduction of these techniques helps to improve the generalization capability and performance of dialogue systems in different domains. Bowden et al. [18] presented a data-driven method through inspecting and cataloging massive amounts of social media information. By fusing sentiment and style analysis, topic modeling, and summarization tasks, they aimed to create a personal assistant with more subtle language comprehension skills. Vakulenko et al. [19] proposed a data-driven model for understanding the structure of information retrieval dialogues. Cuayhuitl et al. [20] introduced SimpleDS, a simple and publicly available dialogue system trained based on deep reinforcement learning, which induces reasonable behavior by increasing the level of dialogue control automation. TodingBunga et al. [21] investigated the approach of building a comprehensive dialogue system using Long Short-Term Memory (LSTM) by combining rule-based and data-driven approaches, achieving high-performance dialogue systems.

However, most dialogue systems focus on general domains and lack adaptation to the financial domain. Therefore, research in the financial domain is a current key point, aiming to address the gap in specialized domains. Therefore, there is a pressing need to study dialogue systems that have strong response capabilities in vertical domains, enabling general-purpose dialogue systems to have high accuracy and robustness in specialized domains. We propose a financial knowledge-enhanced question matching method, suitable for both common and specialized questions in the financial domain, thereby enhancing the accuracy and robustness of the dialogue system.

### 2.2. Text Matching

Text matching is a key task in the field of natural language processing. It involves comparing two or more text segments to determine their similarity or relationship. Many existing text-matching methods tend to utilize deep learning models. Huang et al. [6] introduced the Deep Structured Semantic Model (DSSM), which tackles the challenge of matching queries to relevant documents at the semantic level. It achieves this by projecting queries and documents into a shared low-dimensional space to compute correlations between them. Shen et al. [5] proposed a novel latent semantic model based on convolutional neural networks, enhancing the DSSM model by learning low-dimensional semantic vectors for search queries and web documents. Rao et al. [22] introduced a novel model, HCAN (Hybrid Co-Attention Network), which addresses relevance matching and semantic matching tasks by measuring the semantic distance between two short text segments. Nie et al. [23] proposed a multi-domain natural language inference model called Shortcut-Stacked, specifically addressing the task of natural language inference. Jonas et al. [24] proposed a twin-based Long Short-Term Memory (LSTM) network that assesses the semantic similarity between sentences, overcoming the challenge posed by labeled data consisting of pairs of variable-length sequences. In the medical field, text matching also plays a crucial role. Li et al. [25] proposed a method that combines the word2vec model and TF-IDF, applied within an online medical consultation platform.

Several studies leverage text similarity matching for natural language inference tasks encompassing entailment, contradiction, and neutrality. Zhou et al. [26] proposed a multi-view response selection model that integrates information from distinct perspectives, capturing both discourse-level discourse information and dependencies. Pang et al. [7] introduced a text-matching model treating the problem akin to image recognition. They successfully addressed text-matching challenges in NLP by hierarchically combining patterns, reminiscent of those employed in image recognition techniques. Parikh et al. [27] presented a simple neural architecture that decomposes the problem into subproblems addressable via attention mechanisms. This model facilitates easy parallelization and enhances accuracy in natural language inference tasks. Wang et al. [28] proposed a general “compare-aggregate” framework utilizing convolutional neural networks for word-level matching, followed by aggregation. This approach effectively deals with comparing relationships between different sequences. He et al. [29] introduced a novel similarity attention mechanism explicitly modeling pairwise word interactions to identify crucial correspondences for improved similarity measurement, addressing issues in text similarity. Zhou et al. [30] extended the attention mechanism in two ways: by employing stacked self-attention mechanisms for constructing representations of different granularities of text segments, and by extracting truly matching segment pairs in the attention between context and response. This extension addresses context co-reference relationships in multi-turn dialogues.

With the rapid development of NLP, text-matching techniques are being employed and adapted across various downstream tasks and diverse domains. The logic behind semantic matching tasks involves taking a pair of samples as input and producing an output indicating the similarity relationship between them. Zhao et al. [31] decomposed sentence-level similarity into entity-matching scores and context-matching scores to address semantic matching problems. Web retrieval similarly extensively employs the principles of text matching. Huang et al. [32] proposed a multi-dimensional representation neural network that incorporates TF-IDF, Word2Vec, and ELMo, effectively enhancing the performance of web retrieval. In the field of text summarization, text matching techniques are also utilized to identify duplicate text content, facilitating the removal of redundant information from summaries, which is beneficial for text mining purposes. Mishra et al. [33] proposed an embedded model to examine the similarity of summary texts, effectively addressing this issue. Long-text QA is also an important challenge. Kuang et al. [34] introduced convolutional neural networks to enhance the Enhanced Sequential Inference Model (ESIM), enabling it to better extract features from long texts.

However, most existing text-matching methods perform well in general benchmarks but exhibit poor generalization to the financial domain. These methods heavily rely on domain-specific features and contexts, making them less adaptable to different data distributions and domain knowledge. We propose a financial knowledge-enhanced question-matching model, which not only enhances the accuracy of matching user queries with questions but also improves the generalization capability of text-matching techniques in the financial domain.

## 3. Proposed Method

**Figure 2:**
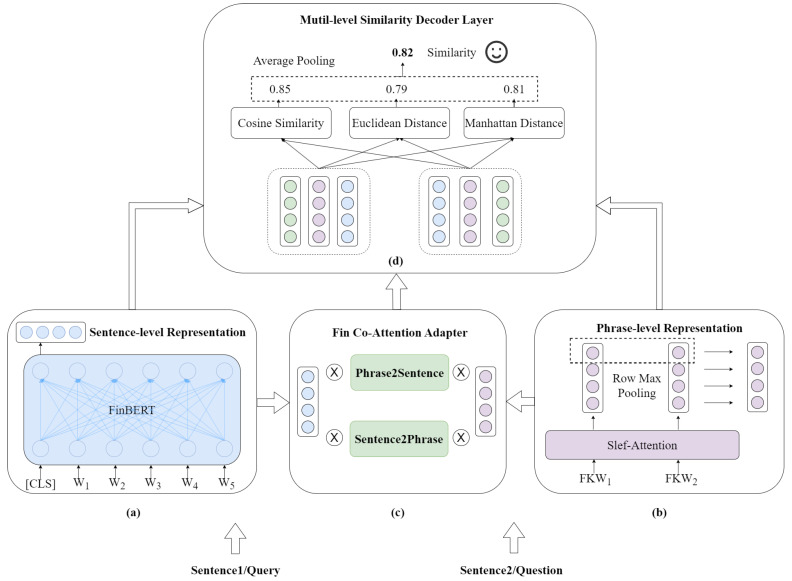
**Illustration of the proposed approach**. (**a**) is sentence-level representation, (**b**) is phrase-level representation, (**c**) is fin co-attention adapter, and (**d**) is similarity decoder layer.

In this section, we introduce the proposed method, as shown in Figure 2. We design the model based on a dual similarity text matching architecture [5,6], consisting of a multi-level encoder layer (Section 3.2), a fin co-attention adapter (Section 3.3), and a multi-level similarity decoder layer (Section 3.4). The multi-level encoder layer ensures the completeness of financial text representation from the views of both sentence and financial phrases. The sentence-level representation is responsible for representing utterance, as shown in Figure 2a. The phrase-level representation is responsible for representing financial phrases, as illustrated in Figure 2b. The fin co-attention adapter is responsible for integrating sentence vectors and financial phrase vectors to generate a comprehensive text representation, as depicted in Figure 2c. The multi-level similarity decoder layer is responsible for computing the text similarity between the representations of query and question, and it outputs the labels, as shown in Figure 2d.

### 3.1. Problem Definition

In this paper, we define model inputs as X={Query,Question} where Query represents the user query, and Question denotes pre-defined questions, FinBERT inputs as $C=(\left[CLS\right],c_{1},\ldots ,c_{n})$, fin-keywords sequence as $FKW=(fkw_{1},\ldots ,fkw_{n})$, and model output label as Y(Y∈0,1). During the training, y=1 signifies similarity between the Query and Question, and y=0 indicates dissimilarity between the Query and Question, as shown in Table 2. The objective of question matching is to accurately distinguish whether a Query and a Question are similar or dissimilar.

### 3.2. Multi-Level Encoder Layer

Due to the specificity of financial domain text, we extract both sentence features and financial phrase features separately. Therefore, we design a multi-level encoder layer, including sentence-level and phrase-level representation. The sentence-level representation aims to encode financial text representations that are inclined to the financial context. The phrase-level representation is able to enhance the adaptability of utterances to financial contexts by directly encoding financial phrases within sentences. We first extract sentence features from the text, and the financial keyword phrase features are incorporated to enhance the text features, thereby improving the representational capabilities of the proposed model. Some examples of Chinese financial phrases are illustrated in Table 1.

#### 3.2.1. Sentence-Level Representation

**Figure 3:**
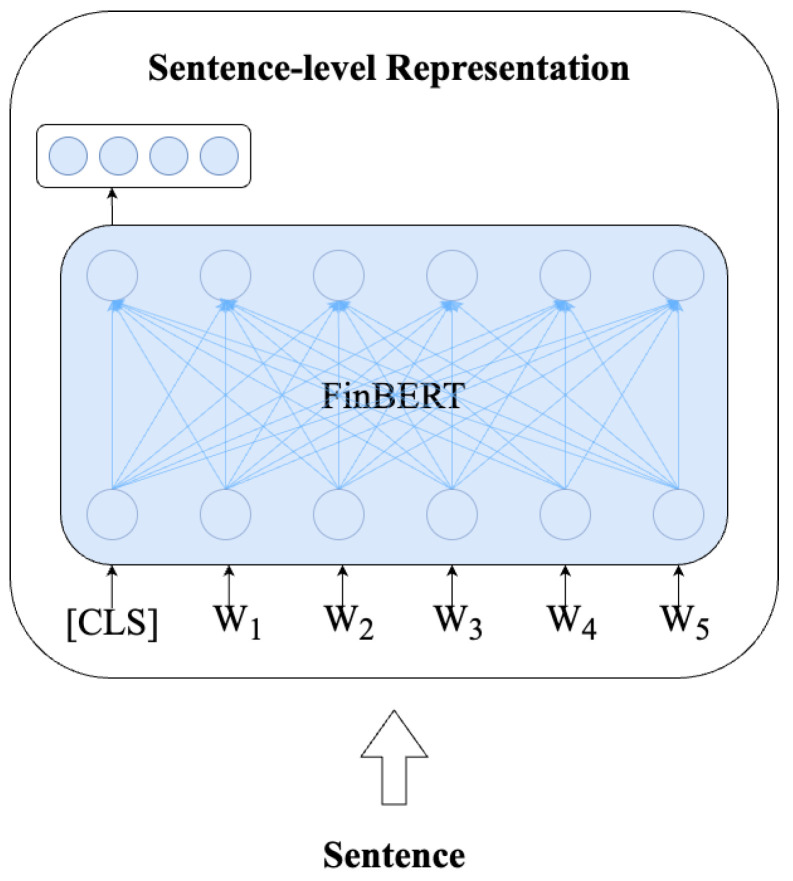
**Illustration of the architecture of the FinBERT.**

We employ the FinBERT [10] model to encode text vectors. The FinBERT is a financial pre-trained language model designed on BERT [35]. Both possess identical model architectures, and the FinBERT workflow is depicted in Figure 3. Its pretraining corpus and tasks are specifically tailored for the financial domain. Therefore, The FinBERT is better suited to represent financial text. The formula is as follows:

(1) $$\begin{array}{cc} \; & H_{query}=FinBERT\left(C_{query}\right),H_{query}\in R^{n\times m}\\ \; & H_{question}=FinBERT\left(C_{question}\right),H_{question}\in R^{n\times m} \end{array}$$

where $C_{query}$ is user query input, $C_{question}$ is qusetion text, $H_{query}$ and $H_{qusetion}$ is output of FinBERT, *n* is length of text, and *m* is hidden layer dimension.

#### 3.2.2. Phrase-Level Representation

Our model enhances the ability of sentence representation by incorporating financial phrases, bolstering the ability to discern financial knowledge.

**Figure 4:**
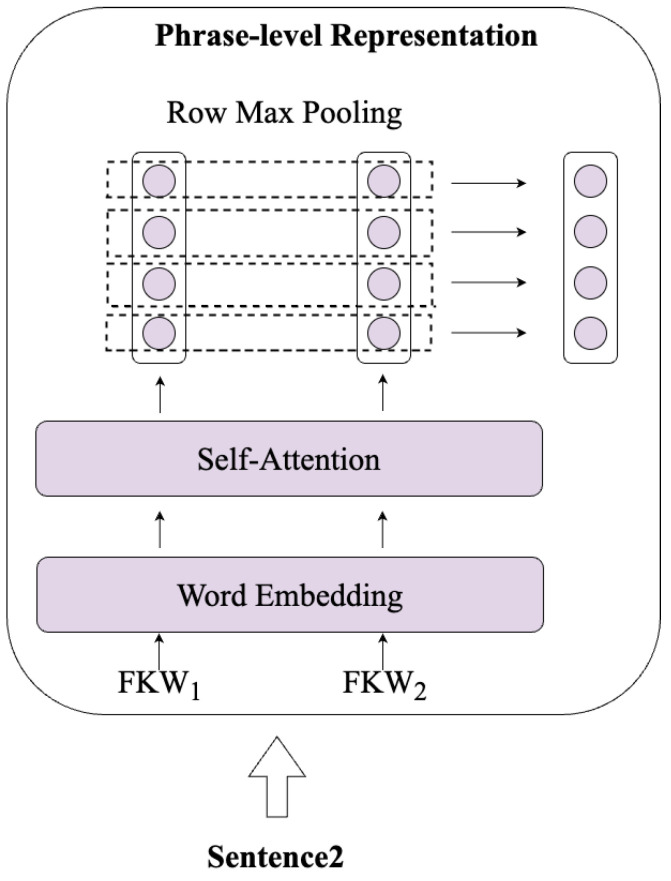
**Illustration of the workflow of the Phrase-level Representation.**

As shown in Figure 4, Firstly, financial phrases are encoded using the Word Embedding, as shown in the following formula:

(2) $$\begin{array}{cc} \; & E_{query}=W_{eb}\left(FKW_{query}\right),E_{query}\in R^{l\times m}\\ \; & E_{qusetion}=W_{eb}\left(FKW_{qusetion}\right),E_{qusetion}\in R^{l\times m} \end{array}$$

where $W_{eb}$ is trainable financial keywords embedding matrix, $FKW_{query}$ is financial phrases of query, $FKW_{qusetion}$ is financial phrases of qusetion, *l* is length of financial keywords, and *m* is hidden layer dimension.

Then, a self-attention layer is utilized to capture the relationships between financial phrases, as follows:

(3) $$\begin{array}{cc} \; & Q_{query}=\sigma \left(E_{query}W_{q}\right),Q_{query}\in R^{l\times m}\\ \; & K_{query}=\sigma \left(E_{query}W_{k}\right),K_{query}\in R^{l\times m}\\ \; & V_{query}=\sigma \left(E_{query}W_{v}\right),V_{query}\in R^{l\times m}\\ \; & O_{query}=softmax\left(Q_{query}K_{query}^{T}\right)V_{query},O_{query}\in R^{l\times m} \end{array}$$

where σ is an activation function, $W_{q}$ is trainable matrix, $W_{k}$ is trainable matrix, $W_{v}$ is trainable matrix, *l* is length of financial keywords, and *m* is hidden layer dimension.

Additionally, our method employs max pooling operations at the row dimension level which ensures different numbers of financial keywords within a sentence are represented in a uniform dimension, as illustrated in the following formula:

(4) $${\bar{O}}_{query}=MaxPool\left(O_{query}\right),{\bar{O}}_{query}\in R^{1\times m}$$

where MaxPool is a row max pooling function, and *m* is hidden layer dimension.

Our model performs the same processing in parallel for the Question, as shown in the following formula:

(5) $$\begin{array}{cc} \; & Q_{qusetion}=\sigma \left(E_{qusetion}W_{q}\right),Q_{qusetion}\in R^{l\times m}\\ \; & K_{qusetion}=\sigma \left(E_{qusetion}W_{k}\right),K_{qusetion}\in R^{l\times m}\\ \; & V_{qusetion}=\sigma \left(E_{qusetion}W_{v}\right),V_{qusetion}\in R^{l\times m}\\ \; & O_{qusetion}=softmax\left(Q_{qusetion}K_{qusetion}^{T}\right)V_{qusetion},O_{qusetion}\in R^{l\times m} \end{array}$$

where σ is an activation function, $W_{q}$ is trainable matrix, $W_{k}$ is trainable matrix, $W_{v}$ is trainable matrix, *l* is length of financial keywords, and *m* is hidden layer dimension.

(6) $${\bar{O}}_{qusetion}=MaxPool\left(O_{qusetion}\right),{\bar{O}}_{qusetion}\in R^{1\times m}$$

where MaxPool is a row max pooling function, and *m* is hidden layer dimension.

### 3.3. Fin Co-Attention Adapter

**Figure 5:**
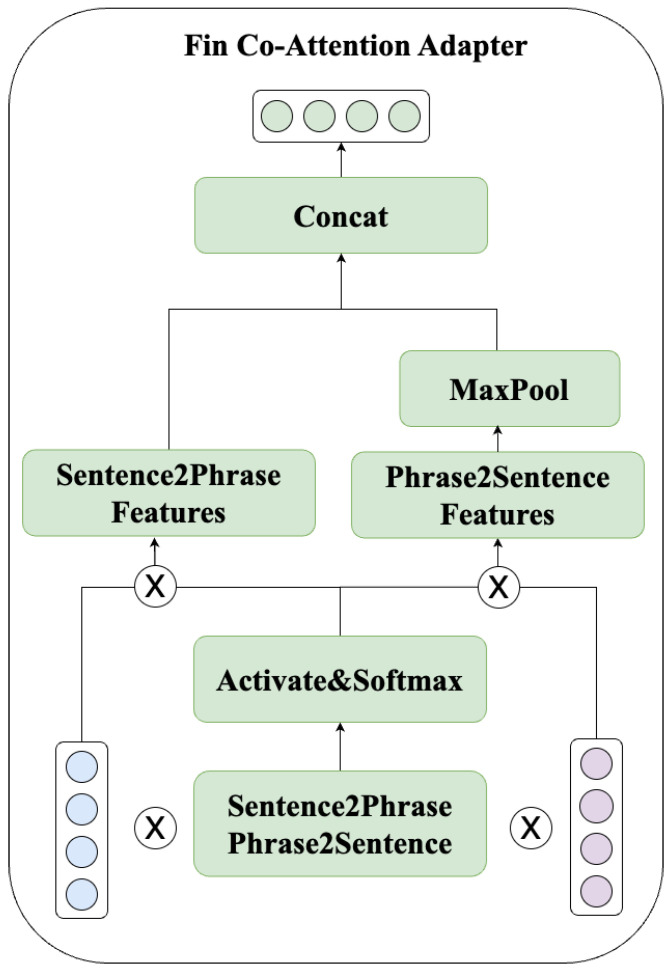
**Illustration of the workflow of the Fin Co-Attention Adapter.**

The proposed model employs a co-attention adapter [22] to combine sentence representations with financial phrase representations to obtain contextual representations. The workflow of this module is illustrated in Figure 5.

Firstly, calculate the co-attention scores from both directions (phrase-to-sentence and sentence-to-phrase), as shown in the following formula:

(7) $$\begin{array}{cc} \; & A=\sigma \left(H_{query}W_{sk}{\bar{O}}_{query}^{T}\right),W_{sk}\in R^{m\times m},A\in R^{n\times 1}\\ \; & \hat{A}=softmax_{col}\left(A\right),\hat{A}\in R^{n\times 1} \end{array}$$

where $W_{sk}$ is trainable matrix, and *m* is hidden layer dimension.

Subsequently, derive the sentence vector representation enriched with financial words and the financial word vector representation enriched with sentences, as represented in the following formulas:

(8) $$\begin{array}{cc} \; & {\hat{H}}_{query}={\hat{A}}^{T}H_{query},{\hat{H}}_{query}\in R^{1\times m}\\ \; & {\hat{O}}_{query}=MaxPool\left(\hat{A}{\bar{O}}_{query}\right),{\hat{O}}_{query}\in R^{1\times m} \end{array}$$

where MaxPool is a row max pooling function, and *m* is hidden layer dimension.

Finally, concatenate ${\hat{H}}_{query}$ and ${\hat{O}}_{query}$ to obtain the contextual representation, as expressed in the following formula:

(9) $$CA_{query}=\left[{\hat{H}}_{query}⊕{\hat{O}}_{query}\right],CA_{query}\in R^{2\times m}$$

where ⊕ is the concatenation operation, and *m* is hidden layer dimension.

For the Question, our method performs the same parallel processing, as described in the following formula:

(10) $$\begin{array}{cc} \; & A=\sigma \left(H_{qusetion}W_{sk}{\bar{O}}_{qusetion}^{T}\right),W_{sk}\in R^{m\times m},A\in R^{n\times 1}\\ \; & \hat{A}=softmax_{col}\left(A\right),A\in R^{n\times 1} \end{array}$$

where $W_{sk}$ is trainable matrix, and *m* is hidden layer dimension.

(11) $$\begin{array}{cc} \; & {\hat{H}}_{qusetion}={\hat{A}}^{T}H_{qusetion},{\hat{H}}_{qusetion}\in R^{1\times m}\\ \; & {\hat{O}}_{qusetion}=MaxPool\left(\hat{A}{\bar{O}}_{qusetion}\right),{\hat{O}}_{qusetion}\in R^{1\times m} \end{array}$$

where MaxPool is a row max pooling function, and *m* is hidden layer dimension.

(12) $$CA_{qusetion}=\left[{\hat{H}}_{qusetion}⊕{\hat{O}}_{qusetion}\right],CA_{qusetion}\in R^{2\times m}$$

where ⊕ is the concatenation operation, and *m* is hidden layer dimension.

### 3.4. Mutil-Level Similarity Decoder Layer

The proposed model uses a multi-level similarity encoder to calculate the similarity between Query and Question, mitigating the randomness associated with a single calculation formula. Our model concatenates CA, $H^{\left[CLS\right]}$, and $\bar{O}$ together to form the final discriminative representation, as expressed in the following formula:

(13) $$\begin{array}{cc} \; & O_{query}^{SD}=\left[CA_{query}⊕H_{query}^{\left[CLS\right]}⊕{\bar{O}}_{query}\right],O_{query}^{SD}\in R^{4\times m}\\ \; & O_{qusetion}^{SD}=\left[CA_{qusetion}⊕H_{qusetion}^{\left[CLS\right]}⊕{\bar{O}}_{qusetion}\right],O_{qusetion}^{SD}\in R^{4\times m} \end{array}$$

where $H^{\left[CLS\right]}$ is the hidden states of the [CLS], and *m* is hidden layer dimension.

Subsequently, similarity scores for $O_{query}^{SD}$ and $O_{question}^{SD}$ are calculated using cosine similarity, Manhattan distance, and Euclidean distance, as illustrated in the following formulas:

**Cosine Similarity**
  (14) $$Dis_{cs}=\frac{O_{query}^{SD}⊙O_{qusetion}^{SD}}{\sqrt{\sum_{i=1}^{n}{\left(O_{query}^{SD}\right)}^{2}}\sqrt{\sum_{i=1}^{n}{\left(O_{qusetion}^{SD}\right)}^{2}}},Dis_{cs}\in \left[0,1\right]$$
  where ⊙ is the dot product operation.

**Manhattan Distance**
(15) $$Dis_{md}=Sigmoid\left(\sum_{i=1}^{n}|O_{query}^{SD}-O_{qusetion}^{SD}|\right),Dis_{md}\in \left[0,1\right]$$

**Euclidean Distance**
(16) $$Dis_{ed}=Sigmoid\left(\sqrt{\sum_{i=1}^{n}{\left(O_{query}^{SD}-O_{qusetion}^{SD}\right)}^{2}}\right),Dis_{ed}\in \left[0,1\right]$$

Finally, our model computes the average of the three discriminative distances as the final model output, as represented in the following formula:

(17) $$Y=Mean\left(Dis_{cs}+Dis_{md}+Dis_{ed}\right),Y\in \left[0,1\right]$$

where *Y* ≥ 0.65 means label is 1, Y<0.65 means label is 0.

**Cross Entropy Loss** The training objective of our method is to minimize the score of L, L is the output of the Cross entropy loss function, as follows:
  (18) $$L=-Y_{true}\log Y-\left(1-Y_{true}\right)\log\left(1-Y\right)$$
  where $Y_{true}$ represents the true labels.

## 4. Experiments

In this section, we introduce the dataset (Section 4.1), for our proposed model and the experimental settings (Section 4.2). Then, we discuss the baseline methods (Section 4.3) and analyze the main experimental results (Section 4.4).

### 4.1. Dataset

We evaluate the proposed methods on a Chinese financial text semantic similarity dataset called the Ant Financial Question Matching Corpus (AFQMC) (https://autonlp.ai/datasets/ant-financial-question-matching-corpus-(afqmc)-(clue-benchmark)) This dataset consists of 102,477 pairs of sentences, consisting of 18,685 positive pairs and 83,792 negative pairs. We divide the dataset into training (0.7), validation (0.2), and test sets (0.1), with the details shown in Table 3. The training set has 71,734 sentence pairs, positive labels have 13,079, negative labels have 58,654, the mean length of positive sentence pairs is 26, and the mean length of negative sentence pairs is 25. The validation set has 20,495 sentence pairs, positive labels have 3737, negative labels have 16,758, the mean length of positive sentence pairs is 25, and the mean length of negative sentence pairs is 25. The test set has 10,248 sentence pairs, positive labels have 1869, negative labels have 8380, the mean length of positive sentence pairs is 26, and the mean length of negative sentence pairs is 25.

### 4.2. Experimental Setups

Table 4 shows the experimental hyper-parameters setting information. Our proposed model uses FinBERT [10] as the sentence-level representation layer, which has 12 layers, 768 hidden states, and 12 heads. The financial keywords embedding dimension is 768. The co-attention dimension is 768, and the batch size is 256. The optimizer for model training uses AdamW [36], with a learning rate of 5 × $10^{-5}$. The dropout probability is 0.1. The different epochs are set as [10, 20, 30, 40, 50, 60, 80]. For the training process, all of the hyper-parameters are tuned on the validation dataset. For the development environment, the CPU is Intel(R)Core(TM)i7-11700F@2.5 GHz, the video card is GeForce RTX 3090 GPU, the operating system is Ubuntu 20, the development tool is Pycharm, the programming language is Python 3.8, and the development framework is Pytorch 2.0.

### 4.3. Baseline Models

To validate the performance of the proposed model, we compare our method with existing state-of-the-art (SOTA) methods, the baselines are as follows:

- **DSSM. Ref. [6]** DSSM is a deep-structured latent semantic model used to model queries and documents.
- **CDSSM. Ref. [5]** CDSSM enhances DSSM with Convolutional Neural Networks (CNN), resulting in a superior semantic model.
- **QACNN. Ref. [37]** QACNN employs multiple deep CNNs to address non-factoid question-answering tasks.
- **QALSTM. Ref. [8]** QALSTM utilizes a hybrid model incorporating both CNN and LSTM for applications in the insurance question-answering domain.
- **DARCNN. Ref. [38]** DARCNN leverages a hybrid model that combines self-attention, cross-attention, and CNN to model the answer selection task.
- **BERT. Ref. [35]** BERT is a pre-trained model based on the Transformer encoder architecture, using the contextual states corresponding to [CLS] to determine text similarity.
- **FinBERT. Ref. [10]** FinBERT is pre-trained based on financial tasks and financial text, which leads to its output context vectors being more inclined to financial contexts.

### 4.4. Experimental Results

Based on previous methods [9], we chose Accuracy (Acc), Recall, and F1 score as the evaluation metrics for the proposed method. Table 5 shows that our proposed model achieves the best results compared to all baseline methods. In particular, compared to FinBERT, the proposed method shows significant improvements with an increase of 1.05% in accuracy (from 73.10% to 74.15%), an increase of 1.69% in recall (from 73.21% to 74.90%), and an increase of 1.37% in F1 score (from 73.16% to 74.53%). Therefore, our proposed model becomes the new state-of-the-art (SOTA) method, and the main reasons are as follows: (1) In the financial context, FinBERT is more effectively attuned to represent utterance context vectors. Furthermore, encoding financial phrases improves the sentence representation capabilities. (2) The financial co-attention adapter integrates both sentence representations and financial phrase representations, enhancing the adaptability of contextual vectors to the financial domain. (3) The multi-level similarity decoder calculates similarity with reduced randomness, thereby enhancing the ability of discriminative.

## 5. Analysis

In this section, we conduct an ablation analysis (Section 5.1) and describe the analysis of the multi-level similarity decoder (Section 5.2). Furthermore, we describe a case study (Section 5.3) of our proposed model.

### 5.1. Analysis of Ablation Experiments

To analyze the interpretability of our proposed model, we conduct ablation experiments as follows:

- **w/o phrase-level rep.** To verify the effectiveness of financial phrase representations in our model, we removed the Phrase-level Representation layer from the proposed model. The model directly uses contextual vectors (from [CLS] of FinBERT) to calculate the similarity between the Query and the Question. The final contextual vectors of $O_{query}^{SD}$ and $O_{qusetion}^{SD}$ are as follows:
  (19) $$\begin{array}{cc} \; & O_{query}^{SD}=H_{query}^{\left[CLS\right]},O_{query}^{SD}\in R^{1\times m}\\ \; & O_{qusetion}^{SD}=H_{qusetion}^{\left[CLS\right]},O_{qusetion}^{SD}\in R^{1\times m} \end{array}$$
  where $H^{\left[CLS\right]}$ is the hidden states of the [CLS], and *m* is hidden layer dimension.
- **w/o fin co-attn.** To validate the effectiveness of the Fin Co-Attention adapter, we removed this module from the proposed model. The final contextual vectors for predicting text similarity are obtained by concatenating the contextual vectors (from [CLS] of FinBERT) and the financial phrase vectors from the Phrase-level Representation layer.
  The formula is as follows:
  (20) $$\begin{array}{cc} \; & O_{query}^{SD}=\left[H_{query}^{\left[CLS\right]}⊕{\bar{O}}_{query}\right],O_{query}^{SD}\in R^{2\times m}\\ \; & O_{qusetion}^{SD}=\left[H_{qusetion}^{\left[CLS\right]}⊕{\bar{O}}_{qusetion}\right],O_{qusetion}^{SD}\in R^{2\times m} \end{array}$$
  where $H^{\left[CLS\right]}$ is the hidden states of the [CLS], and *m* is hidden layer dimension.

Table 6 shows the results of our proposed model with one or more modules removed. After removing modules, there is a significant decline in all performance metrics. Because specific financial key phrases can enhance the ability of sentence context representations in the financial domain. Removing the module of phrase-level representation results in the inability to learn the features of specific financial key phrases. The result evidences that the phrase-level representation module enhances the ability of the proposed model to recognize specific financial phrases. Removing the fin co-attention layer results in the simple concatenation of sentence representations and financial phrase representations, leading to a reduction in the learning capability of the model. The result provides evidence that the Co-Attention Adapter helps the model capture the relationship between sentence representations and financial phrase representations. Therefore, the proposed modules all contribute to improving the predictive performance. Further, demonstrates the interpretability of the proposed model.

### 5.2. Analysis of Multi-Level Similarity Decoder

To validate the effectiveness of the multi-level similarity decoder, we design the following experiments:

- **single Cosine Similarity (sCS).** This layer only employs cosine similarity to calculate the similarity between Query and Question. The process of model prediction for the label *Y* is as follows:
  (21) $$Y=Dis_{cs},Y\in \left[0,1\right]$$
  where *Y* ≥ 0.65 means label is 1, Y<0.65 means label is 0.
- **single Manhattan Distance (sMD).** This layer only employs Manhattan distance to calculate the similarity between Query and Question. The process of model prediction for the label *Y* is as follows:
  (22) $$Y=Dis_{md},Y\in \left[0,1\right]$$
  where *Y* ≥ 0.65 means label is 1, Y<0.65 means label is 0.
- **single Euclidean Distance (sED).** This layer only employs Euclidean distance to calculate the similarity between Query and Question. The process of model prediction for the label *Y* is as follows:
  (23) $$Y=Dis_{ed},Y\in \left[0,1\right]$$
  where *Y* ≥ 0.65 means label is 1, Y<0.65 means label is 0.
- **CS + MD.** This layer uses the average of cosine similarity and Manhattan distance to calculate the similarity between Query and Question. The process of model prediction for the label *Y* is as follows:
  (24) $$Y=Mean\left(Dis_{cs}+Dis_{md}\right),Y\in \left[0,1\right]$$
  where *Y* ≥ 0.65 means label is 1, Y<0.65 means label is 0.
- **CS + ED.** This layer uses the average of cosine similarity and Euclidean distance to calculate the similarity between Query and Question. The process of model prediction for the label *Y* is as follows:
  (25) $$Y=Mean\left(Dis_{cs}+Dis_{ed}\right),Y\in \left[0,1\right]$$
  where *Y* ≥ 0.65 means label is 1, Y<0.65 means label is 0.
- **MD + ED.** This layer uses the average of Manhattan distance and Euclidean distance to calculate the similarity between Query and Question. The process of model prediction for the label *Y* is as follows:
  (26) $$Y=Mean\left(Dis_{md}+Dis_{ed}\right),Y\in \left[0,1\right]$$
  where *Y* ≥ 0.65 means label is 1, Y<0.65 means label is 0.
- **CS + MD + ED.** This layer uses the average of cosine similarity, Manhattan distance, and Euclidean distance to calculate the similarity between Query and Question. The process of model prediction for the label *Y* is as follows:
  (27) $$Y=Mean\left(Dis_{cs}+Dis_{md}+Dis_{ed}\right),Y\in \left[0,1\right]$$
  where *Y* ≥ 0.65 means label is 1, Y<0.65 means label is 0.

**Figure 6:**
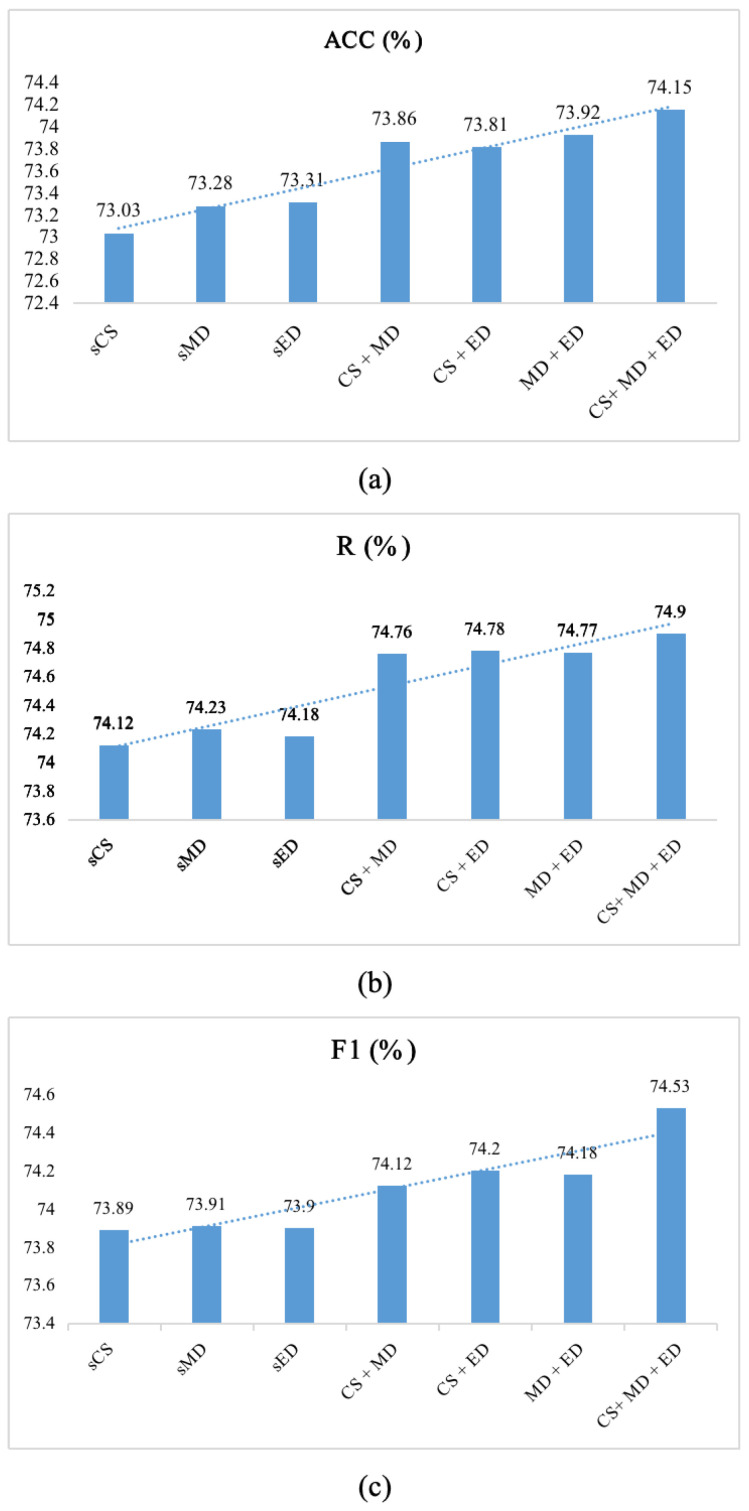
**Analysis of Mutil-level Similarity.** (**a**) is accuracy, (**b**) is recall, and (**c**) is F1 score.

Table 7 shows the results of using one or more similarity calculation methods. We observe that using a single similarity calculation as the similarity decoding layer resulted in the worst model performance. Using a combination of two similarity calculations as the similarity decoding layer leads to some improvement in performance. To facilitate a better understanding of the results, we separately plot trend charts for ACC, Recall, and F1 scores. According to Figure 6, different similarity calculation methods impact the final prediction performance. The results indicate that choosing three methods as the similarity decoder yields the most robust model.

### 5.3. Analysis of Case Study

To compare the inference performance of our proposed model with other models, we design a case study experiment on the DSSM [6] and the proposed method. The Query is “花呗怎么用?”, and the Question is “蚂蚁花呗如何开通?”. Table 8 shows that DSSM made an incorrect prediction, whereas our proposed model made a correct prediction. Because DSSM cannot accurately identify Chinese financial phrases, our proposed model can effectively model financial context representations. Utilizing representation vectors with financial context can more accurate predictions, further demonstrating the adaptability of our model to the financial domain.

### 5.4. Analysis of Effectiveness of Model in Different Language

To verify the generalizability and scalability of the proposed method, we design experiments on new financial text-matching datasets in other languages. Our experiments are based on an English dataset, a translated version derived from AFQMC using the ChatGPT API. The Table 9 demonstrates significant improvements in our method’s Acc, Recall, and F1 scores. Specifically, the proposed model on the AFQMC-English dataset shows an increase from 72.76% to 73.78% in Acc, from 72.86% to 73.87% in Recall, and from 72.93% to 73.86% in F1 score. The experimental results further demonstrate the generalizability and scalability of our proposed method.

### 5.5. Model Result Analysis

**Figure 7:**
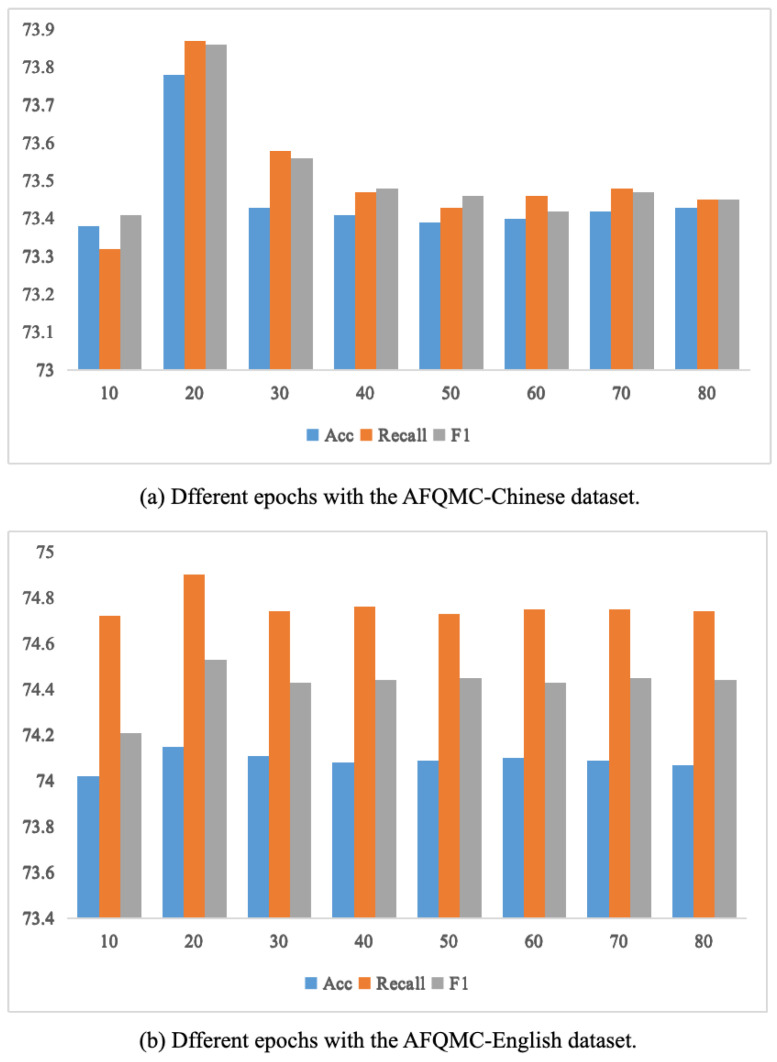
**Analysis with different epochs.** The epochs are represented on the X-axis, and the scores of Acc, Recall, and F1 are represented on the Y-axis.

To select the optimal results from the dataset, we analyze the performance of the proposed method across different epochs (10, 20, 30, 40, 50, 60, 70, and 80). According to Figure 7a, on the AFQMC-Chinese dataset, the best ACC achieved is 74.15%, the highest Recall is 74.9%, and the peak F1 score is 74.53%. These results are observed at the 20th epoch. As illustrated in Figure 7b, on the AFQMC-English dataset, the highest ACC reaches 73.78%, the peak Recall is 73.87%, and the best F1 score is 73.86%, occurring at the 20th epoch.

## 6. Conclusions, Limitations and Future Work

In this paper, we propose a novel financial knowledge-enhanced network for financial question matching. The proposed method includes a multi-level encoder layer that can extract contextual sentence representations and financial phrase representations, a fin co-attention adapter that can integrate sentence representations and financial phrase representations, forming context representations biased toward the financial domain, and a multi-level similarity decoder layer that can robustly calculate text similarity. Experimental results on the AFQMC dataset show that our proposed model achieves significant improvements in ACC score, Recall score, and F1 score compared to the previous SOTA models. This work contributes to guiding financial text-matching problems; however, it still has some limitations. Our model architecture primarily involves keywords in the financial domain and may not adapt well to key phrases in non-financial domains. It could potentially impact the robustness of the proposed model. Due to the substantial manpower and financial resources required for gathering and organizing text-matching datasets in the financial domain, we validated the effectiveness of our model using datasets in both Chinese and English. It may have an impact on the model’s generalization ability. In the future, we continue to explore question matching in the financial domain through the following two aspects: (1) Expanding the training dataset with a more comprehensive collection of financial texts can enhance the ability to handle a wider range of financial topics. (2) Leveraging the strong language understanding capabilities of large language models can help the model further improve its performance in financial contexts.

## Figures and Tables

**Table 1:** **Examples of Chinese financial keywords.** The left column is the Chinese financial keyword, right column is the corresponding English explanation.

Chinese	English
借呗	Ant Cash Now
花呗	Ant Checklater
余额宝	Alibaba’s Yu’E Bao
收钱码	Payment Code
芝麻信用	Zhima Credit
逾期	Overdue
双十一	Double Eleventh Day
微信红包	WeChat Red Packet

**Table 2:** **Illustration of mathematical symbols.**

Symbol Definition	Description
X={Query,Qusetion}	model inputs
Query	user query
Question	pre-defined questions
*C*	FinBERT inputs
FKW	Fin-keywords sequence
*Y*	model output

**Table 3:** **Detals of Ant Financial Question Matching Corpus (AFQMC) dataset.**

	Training	Validation	Test
Number of sentence-pairs	71,734	20,495	10,248
Positive labels	13,079	3737	1869
Negative labels	58,654	16,758	8380
Mean length of positive sentence-pairs	26	25	26
Mean length of negative sentence-pairs	25	25	25

**Table 4:** **Experimental Setups.**

Parameter Name	Size
FinBERT Hadden Dimension	768
FinBERT Attention Layers	12
FinBERT Attention Heads	12
Financial Keywords Embedding	768
Co-Attention Dimension	768
Dropout	0.1
Batch size	256
Number of epochs	20
Learning rate	5 × $10^{-5}$

**Table 5:** **Main experimental Results.** Acc is accuracy, R is recall, and F1 is F1 score. The results chose the mean of the results obtained from the test set over 10 runs with different random seeds. Additionally, the complexity comprises 120 million trainable parameters. Moreover, after 10 inference iterations, the average inference time for our proposed method is 2.4 ms.

Model	Acc (%)	R (%)	F1 (%)
DSSM [6]	70.38	69.89	70.25
CDSSM [5]	70.52	70.39	70.87
QACNN [37]	69.76	70.07	69.78
QALSTM [8]	70.31	70.96	70.83
DARCNN [38]	71.21	71.43	71.42
BERT [35]	72.04	72.53	72.50
FinBERT [10]	73.10	73.21	73.16
**FinKENet (Ours)**	**74.15**	**74.90**	**74.53**

**Table 6:** **Ablation experiments.** The results chose the mean of the results obtained from the test set over 10 runs with different random seeds.

Model	Acc (%)	R (%)	F1 (%)
w/o phrase-level rep	71.67	71.86	71.92
w/o fin co-attn	72.81	73.11	72.96
**FinKENet (Ours)**	**74.15**	**74.90**	**74.53**

**Table 7:** **Experiments of different combinations of similarity calculations.** The results chose the mean of the results obtained from the test set over 10 runs with different random seeds.

Model	Acc (%)	R (%)	F1 (%)
sCS	73.03	74.12	73.89
sMD	73.28	74.23	73.91
sED	73.31	74.18	73.90
CS + MD	73.86	74.76	74.12
CS + ED	73.81	74.78	74.20
MD + ED	73.92	74.77	74.18
**CS + MD + ED**	**74.15**	**74.90**	**74.53**

**Table 8:** **Case Study.** “花呗怎么用?” is “How to use the Ant Checklate?”, and “蚂蚁花呗如何开通?” is “The Ant Checklate how to open?”

Model	Query/Question	Label
DSSM	花呗怎么用?/ 蚂蚁花呗如何开通?	0
**FinKENet (Ours)**	花呗怎么用?/ 蚂蚁花呗如何开通?	1

**Table 9:** **Experimental Results in AFQMC-english.** Acc is accuracy, R is recall, and F1 is F1 score. The results chose the mean of the results obtained from the test set over 10 runs with different random seeds. Additionally, the complexity comprises 120 million trainable parameters. Moreover, after 10 inference iterations, the average inference time for our proposed method is 2.4 ms.

Model	Acc (%)	R (%)	F1 (%)
DSSM [6]	70.16	69.75	70.14
CDSSM [5]	70.36	70.17	70.64
QACNN [37]	69.53	69.96	69.53
QALSTM [8]	70.17	70.73	70.68
DARCNN [38]	71.06	71.27	71.26
BERT [35]	71.84	72.37	72.39
FinBERT [10]	72.76	72.86	72.93
**FinKENet (Ours)**	**73.78**	**73.87**	**73.86**

## Data Availability

No new data were created or analyzed in this study.
